# Diet meets biologics: accelerating remission in pediatric Crohn’s disease – a real-world experience

**DOI:** 10.3389/fgstr.2026.1951489

**Published:** 2026-10-28

**Authors:** Jhoanna Valery Adauto Luizaga, María Soledad Arcucci, Katia Anabel Kulay, Flavia Vollweiler, Julieta Gallo, Vanina Pagotto, Verónica Busoni, Marina Orsi

**Affiliations:** Pediatric Gastroenterology, Hepatology and Nutrition, Hospital Italiano de Buenos Aires, Buenos Aires, Argentina

**Keywords:** biological therapy, elimination diets, inflammatory bowel disease, nutrition therapy, pediatric Crohn’s disease

## Abstract

**Background:**

Exclusive enteral nutrition (EEN) and the Crohn’s disease exclusion diet (CDED) plus partial enteral nutrition (PEN) induce remission in pediatric Crohn’s disease (CD), but data on their use as adjuncts to biologic therapy remain limited. We aimed to determine whether CDED+PEN combined with first-line biologic therapy reduces the time to clinical and endoscopic remission, framed by STRIDE-II targets, compared with biologics alone.

**Methods:**

Single-center, retrospective cohort study of pediatric patients with CD diagnosed per the revised Porto criteria who initiated first-line anti–tumor necrosis factor-α (anti–TNF-α) therapy between January 2020 and December 2024. Patients with significant comorbidities, inconsistent follow-up, or a change in biologic target were excluded. Group 1 (G1) received CDED+PEN (Modulen-IBD^®^) alongside biologics with multidisciplinary support; Group 2 (G2) received biologics alone. Outcomes were time to clinical response and to clinical remission, both defined by the weighted Pediatric Crohn’s Disease Activity Index (wPCDAI); time to normalization of erythrocyte sedimentation rate (ESR) and C-reactive protein (CRP), time to fecal calprotectin (FC) normalization, and time to mucosal healing (MH). Absolute changes from baseline (Δ) in wPCDAI, FC, Simple Endoscopic Score for CD (SES-CD), ESR, CRP, albumin and body mass index (BMI) were compared between groups, and effect modification by disease location was assessed using median regression models including a diet-by-location interaction term. Between-group comparisons used the Mann–Whitney U test; time-to-event outcomes were analyzed using Kaplan–Meier methods with log-rank comparison.

**Results:**

Forty patients were included (65% male; median age 12.02 years, interquartile range [IQR] 9.52–14.09): 27 in G1 and 13 in G2. Times to clinical response, clinical remission, and ESR/CRP normalization were consistently shorter in G1 without reaching statistical significance. FC normalization was achieved in 72.5% of patients overall and MH in 55.0%. Kaplan–Meier analysis showed no significant difference between groups in the cumulative incidence of either outcome (log-rank p = 0.82 for FC normalization and p = 0.54 for MH). At the prespecified time points, cumulative incidence of FC normalization at 4 months was 23.7% (G1) vs 30.8% (G2), and of MH at 12 months 16.0% (G1) vs 18.2% (G2). Among the absolute changes from baseline, wPCDAI was the only variable showing a significantly greater reduction in G1 (p = 0.029); Δ FC, SES-CD, ESR, CRP, albumin and BMI did not differ between groups. Disease location did not modify the effect of CDED+PEN on any outcome (all p for interaction > 0.10), although the small number of patients with isolated colonic disease (n = 10) renders this analysis exploratory.

**Conclusions:**

In this real-world pediatric cohort, adding CDED+PEN to first-line biologic therapy was associated with a significantly greater reduction in wPCDAI, but not with improved cumulative incidence of FC normalization or MH. The incremental value of adjunctive dietary therapy in children already receiving a biologic may lie in earlier symptomatic control rather than in accelerated resolution of objective inflammation.

## 1 Introduction

Crohn’s disease (CD) is a chronic, relapsing inflammatory bowel disease that affects the gastrointestinal tract in a transmural and discontinuous fashion (4). Pediatric-onset disease tends to be more extensive, to progress more often to stricturing or penetrating behavior, and to carry the added burden of impaired growth, features that prompted a dedicated pediatric classification (12). Diagnosis follows the revised Porto criteria (11), with disease extent and behavior classified according to the Paris classification (12). Anti–TNF-α therapy is widely used in this setting and achieves mucosal healing in a substantial though incomplete proportion of children (17, 18), a context in which the role of nutritional therapy as an adjunct remains poorly defined.

Several nutritional strategies have been evaluated for inducing remission in pediatric patients with Crohn’s disease (CD). Exclusive enteral nutrition (EEN) is an established and effective therapy for inducing deep remission in pediatric patients with low-risk Crohn’s disease, including mucosal healing, owing to its multiple beneficial effects on intestinal health (1–3). However, adherence to EEN remains a significant challenge (4).

More recently, uncontrolled clinical studies have suggested that alternative dietary approaches, including partial enteral nutrition (PEN) and newly developed exclusion diets, may be effective for inducing remission at disease onset (2–4).

The Crohn’s disease exclusion diet (CDED) is a whole-food–based dietary approach designed to reduce exposure to potentially pro-inflammatory dietary components. It aims to limit intake of factors that may adversely affect the gut microbiota, intestinal barrier function, and mucosal immunity, thereby promoting intestinal homeostasis (5, 6). The combination of CDED with PEN has been shown to induce sustained remission in a higher proportion of patients compared with EEN alone, likely driven by improved adherence (6).

Despite the growing body of evidence supporting the effectiveness of CDED plus PEN (5–9), data on its use in combination with other therapeutic strategies, such as biologic therapy, remain limited.

Therefore, the aim of this study was to evaluate whether the addition of CDED combined with PEN to first-line biologic therapy in pediatric patients with CD reduces the time to achieve the therapeutic targets defined by the Selecting Therapeutic Targets in Inflammatory Bowel Disease (STRIDE-II) consensus—clinical response and remission as immediate and intermediate targets, normalization of ESR, CRP and FC as intermediate targets, and endoscopic healing as a long-term target (10)—compared with biologic therapy alone.

## 2 Materials and methods

### 2.1 Study design and population

We conducted a single-center, retrospective cohort study at a national referral center for pediatric inflammatory bowel disease in Argentina. Eligible participants were patients under 18 years of age with a confirmed diagnosis of CD based on the revised Porto criteria (11), who initiated first-line anti–TNF-α therapy between January 1, 2020, and December 31, 2024. Clinical, demographic, laboratory, and treatment-related variables were extracted from medical records at diagnosis, at biologic initiation, and at the time of data collection. Baseline demographic and clinical characteristics, including disease location, behavior, perianal disease, and growth impairment, were classified according to the Paris classification (12).

Patients were excluded if any of the following criteria were met: presence of significant comorbidities, including prior intestinal resection, underlying chronic diseases, or gastrointestinal motility disorders; inconsistent clinical follow-up, defined as missing more than two scheduled visits during the study period; or optimization and change of biologic therapy during the study period.

At the time of diagnosis, patients for whom biologic therapy was indicated could be offered the CDED combined with PEN as an initial intervention. This approach was not standardized and was based on clinician judgment and patient preference.

Anti-TNF therapy was administered according to standard weight-based dosing protocols: infliximab 5 mg/kg at weeks 0, 2, and 6, then every 8 weeks; adalimumab 160/80 mg loading dose (or 80/40 mg for patients weighing <40 kg), followed by 40 mg (or 20 mg) every 2 weeks.

Eligible patients were categorized into two groups according to dietary exposure during induction: Group 1 (G1), patients who received the CDED combined with PEN in addition to biologic therapy (anti–TNF-α); and Group 2 (G2), patients who received biologic therapy alone, without a structured dietary intervention.

### 2.2 Dietary intervention

Patients in G1 received the CDED combined with PEN alongside biologic therapy, following the three-phase structure of the published protocol (5, 6).

Phase 1 (weeks 0–6) constitutes the induction phase and is the most restrictive. Approximately 50% of daily caloric requirements were provided by PEN (Modulen-IBD^®^), with the remainder derived from whole foods permitted in this phase. The diet is built around a set of mandatory daily foods—chicken breast, eggs, potato, banana and apple—intended to ensure adequate intake of protein, resistant starch and pectin, and it allows a defined list of additional fruits, vegetables and rice. It excludes components hypothesized to impair barrier function and promote dysbiosis, including gluten-containing grains, dairy products, animal and processed fats, emulsifiers, maltodextrins, carrageenan, sulfites, canned and processed foods, and red and processed meat.

Phase 2 (weeks 7–12) maintains the same exclusions while progressively reintroducing additional whole foods, including further fruits, vegetables, legumes and selected grains, with a reduction of PEN to approximately 25% of daily caloric requirements.

Phase 3 constitutes the maintenance phase and begins at week 13. It permits a broader range of foods on most days while preserving the core exclusions, with PEN maintained at approximately 25% of caloric requirements. In the present cohort, patients continued in phase 3 beyond the 24 weeks of the original protocol and remained on the diet for the duration of follow-up, rather than returning to a free diet at week 24. The median duration of the dietary intervention was therefore substantially longer than the induction and consolidation phases alone (see Results). Adherence to the dietary intervention was supported through a structured multidisciplinary program involving a pediatric gastroenterologist and an IBD-specialized dietitian. Patients attended in-person visits at treatment initiation, week 6, and week 12. Additional support was available between visits via telephone or email to address questions and facilitate adherence. Patients in G2 received anti–TNF therapy according to standard clinical practice, along with general nutritional counseling, without implementation of a structured exclusion diet or use of partial enteral nutrition. No validated adherence questionnaire was used, reflecting the real-world nature of the study.

### 2.3 Outcomes

The outcomes were: time to clinical response (reduction in weighted Pediatric Crohn’s Disease Activity Index [wPCDAI] ≥17.5 points from baseline); time to biochemical remission (simultaneous normalization of ESR <20 mm/h and CRP <0.5 mg/dL) this outcome was analyzed only in patients with abnormal ESR or CRP at baseline; time to clinical remission (wPCDAI <12.5); time to normalization of fecal calprotectin (FC <250 µg/g on two consecutive measurements ≥4 weeks apart); and time to mucosal healing (first ileocolonoscopy showing SES-CD ≤3 or absence of ulcers in any segment).

In addition, absolute changes from baseline (Δ) were evaluated for wPCDAI, FC, SES-CD, ESR, CRP, albumin, and BMI. Mucosal response was defined as a ≥50% reduction in SES-CD from baseline to follow-up endoscopy. These changes were compared between treatment groups and further examined according to disease location to assess whether the effect of CDED differed between patients with ileal involvement and those with isolated colonic disease. An interaction term between treatment group and disease location was included to formally assess effect modification by disease location.

### 2.4 Statistical analysis

Continuous variables were described as median and interquartile range (IQR), and categorical variables as absolute frequencies and percentages. Baseline characteristics were compared between groups using the Mann–Whitney U test for continuous variables and the chi-square or Fisher’s exact test for categorical variables, as appropriate.

For continuous outcomes (wPCDAI, fecal calprotectin, ESR, CRP, albumin, SES-CD, and BMI), within-group changes from baseline to follow-up were assessed using the Wilcoxon signed-rank test for paired observations. Absolute changes from baseline (Δ), calculated as the follow-up value minus the baseline value, were compared between groups using the Mann–Whitney U test.

To assess whether disease location modified the association between CDED and changes in clinical and biochemical outcomes, median regression models (quantile regression, τ = 0.50) were fitted including CDED, disease location, and their interaction term (CDED × disease location). The p value for the interaction term was used to assess effect modification by disease location.

Time-to-event outcomes, including time to fecal calprotectin normalization and time to mucosal healing, were analyzed using Kaplan–Meier methods. Cumulative incidence was estimated as 1 minus the Kaplan–Meier survival probability. Patients without the event at the end of follow-up were censored at the date of their last available assessment. Kaplan–Meier curves were compared between CDED groups using the log-rank test. Cumulative probabilities were estimated at prespecified time points selected according to clinical relevance and the number of patients remaining at risk.

A p value <0.05 was considered statistically significant. Statistical analyses were performed using R software version 4.3.3.

## 3 Results

A total of 40 pediatric patients with CD were included in the final analysis. The cohort was predominantly male (65%), with a median age at inclusion of 12.02 years (IQR 9.52 -14.09). Patients were stratified into two groups according to treatment received: 27 patients (67.5%) received combined therapy with CDED plus PEN alongside biologic treatment (G1), while the remaining 13 patients (32.5%) received biologic therapy alone (G2). The median follow-up duration—defined as time from biologic initiation to study data cutoff or diet discontinuation (whichever occurred first)—was 20.48 months (IQR 13.10 – 36.56) for the entire cohort: 21.21 months (IQR 13.75 – 32.89) in G1 and 15.15 months (IQR 9.48 – 42.46) in G2 (p = 0.718).

A comparison of the baseline demographic and clinical characteristics between the two groups prior to the start of treatment is presented in **Table 1**. While most characteristics were similar between groups, patients in G2 had significantly higher albumin levels at baseline (p=0.006).

**Table 1:** Baseline characteristics of pediatric patients with Crohn’s disease.

Characteristic	Overall (N = 40)	G1 (n = 27)	G2 (n = 13)	p-value
Age at diagnosis (years), median [IQR]	12.02 [9.52, 14.09]	11.70 [9.52, 14.17]	12.57 [10.23, 13.93]	0.675
Disease duration (years), median [IQR]	0.28 [0.16, 0.49]	0.25 [0.12, 0.44]	0.33 [0.16, 0.57]	0.394
Disease location				
L1 (%)	2 (5)	2 (7.4)	0 (0.0)	0.999
L2 (%)	17 (42.5)	13 (48.1)	4 (30.8)	0.333
L3 (%)	20 (50)	12 (44.4)	8 (61.5)	0.501
L4a (%)	22 (55)	14 (51.9)	8 (61.5)	0.737
L4b (%)	2 (5)	1 (3.7)	1 (7.7)	0.999
Perianal Disease (%)	4 (10)	3 (11.1)	1 (7.7)	0.999
Disease location (dichotomous)				
Colonic (L2) (%)	10 (25)	8 (29.6)	2 (15.4)	0.451
Ileal (L1/L3/L4) (%)	30 (75)	19 (70.4)	11 (84.6)	
Disease behavior				
Inflammatory B1 (%)	39 (97.5)	26 (96.3)	13 (100)	1.000
Stricturing/penetrating B2/B3 (%)	1 (2.5)	1 (3.7)	0 (0.0)	0.999
Growth Impairment G1 (%)	13 (32.5)	8 (29.6)	5 (38.5)	0.722
Time from diagnosis to biologic therapy (weeks) median [IQR]	12.07 [3.11, 25.39]	12.29 [3.07, 32.36]	14.86 [6.57, 22.86]	0.919
wPCDAI at diagnosis median [IQR]	45.00 [40.00, 47.00]	45.00 [41.00, 49.00]	42.00 [40.00, 45.00]	0.083
Hematocrit (%) at initiation median [IQR]	35.25 [31.95, 38.00]	34.60 [30.90, 37.25]	37.60 [33.00, 38.00]	0.271
ESR (mm/h) at initiation median [IQR]	27.00 [16.75, 46.00]	36.00 [19.00, 57.00]	18.00 [13.00, 27.00]	0.051
CRP (mg/L) at initiation median [IQR]	9.00 [4.00, 29.52]	11.90 [5.50, 33.00]	5.00 [3.00, 12.40]	0.091
Albumin (g/dL) at diagnosis median [IQR]	3.70 [3.27, 3.92]	3.50 [3.10, 3.80]	3.80 [3.70, 4.00]	0.006
Fecal calprotectin (µg/g) at initiation median [IQR]	1000.00 [827.75, 1022.00]	1000.00 [968.50, 1022.00]	1000.00 [519.00, 1000.00]	0.177
Concomitant treatment at biologic initiation (%)	12 (30.0)	8 (29.6)	4 (30.8)	0.999
Azathioprine (%)	9 (22.5)	6 (22.2)	3 (23.1)	N/A
Methotrexate (%)	2 (5)	1 (3.7)	1 (7.7)	N/A
6-mercaptopurine (%)	1 (2.5)	1 (3.7)	0 (0.0)	N/A
Anti-TNF agent				
Infliximab (%)	13 (32.5)	9 (33.3)	4 (30.8)	0.999
Adalimumab (%)	27 (67.5)	18 (66.7)	9 (69.2)	

Median; IQR, interquartile range; wPCDAI, weighted Pediatric Crohn’s Disease Activity Index; ESR, erythrocyte sedimentation rate; CRP, C-reactive protein.

Among patients in G1, all patients who initiated the diet maintained it until the study data cutoff date; therefore, the duration of dietary intervention was at least as long as the follow-up period in G1 (median 21.21 months, IQR 13.75–32.89), as assessed during scheduled follow-up visits with the nutrition specialist.

The times to clinical response, clinical remission, and ESR/CRP normalization are presented in **Table 2**. Although no statistically significant differences were observed, there was a consistent trend toward shorter times in G1 for all three outcomes. The median time to ESR/CRP normalization was calculated after excluding patients who already met the definition of biochemical remission at baseline (ESR <20 mm/h and CRP <0.5 mg/dL; n=7: n=3 in the G2 and n=4 in G1). Time to fecal calprotectin normalization and mucosal healing were not achieved by all patients during follow-up and are therefore reported separately.

**Table 2:** Time (weeks) to clinical response, clinical remission, and inflammatory marker normalization.

Outcome	G1 (n = 27)	G2 (n = 13)	p-value
Time to clinical response, median [IQR]	4.43 [3.07, 8.43]	6.29 [4.14, 8.00]	0.470
Time to clinical remission, median [IQR]	11.57 [6.14, 19.79]	13.71 [9.14, 18.57]	0.312
Time to ESR/CRP normalization, median [IQR]	10.00 [4.60, 18.00]	11 [8.14, 19]	0.583

IQR, interquartile range; ESR, erythrocyte sedimentation rate; CRP, C-reactive protein.

Fecal calprotectin normalization was achieved in 29/40 patients (72.5%): 19/27 (70.4%) in G1 and 10/13 (76.9%) in G2. Because normalization of calprotectin was achieved at different time points during follow-up, time-to-event analysis was performed using Kaplan–Meier estimates. Kaplan–Meier curves (**Figure 1**) showed no significant difference in the cumulative incidence of fecal calprotectin normalization between G1 and G2 during follow-up (log-rank p = 0.82). At the prespecified 4-month time point, the cumulative incidence of normalization was 30.8% (95% CI 0.5–51.8%) in G2 and 23.7% (95% CI 5.1–38.7%) in G1.

**Figure 1:**
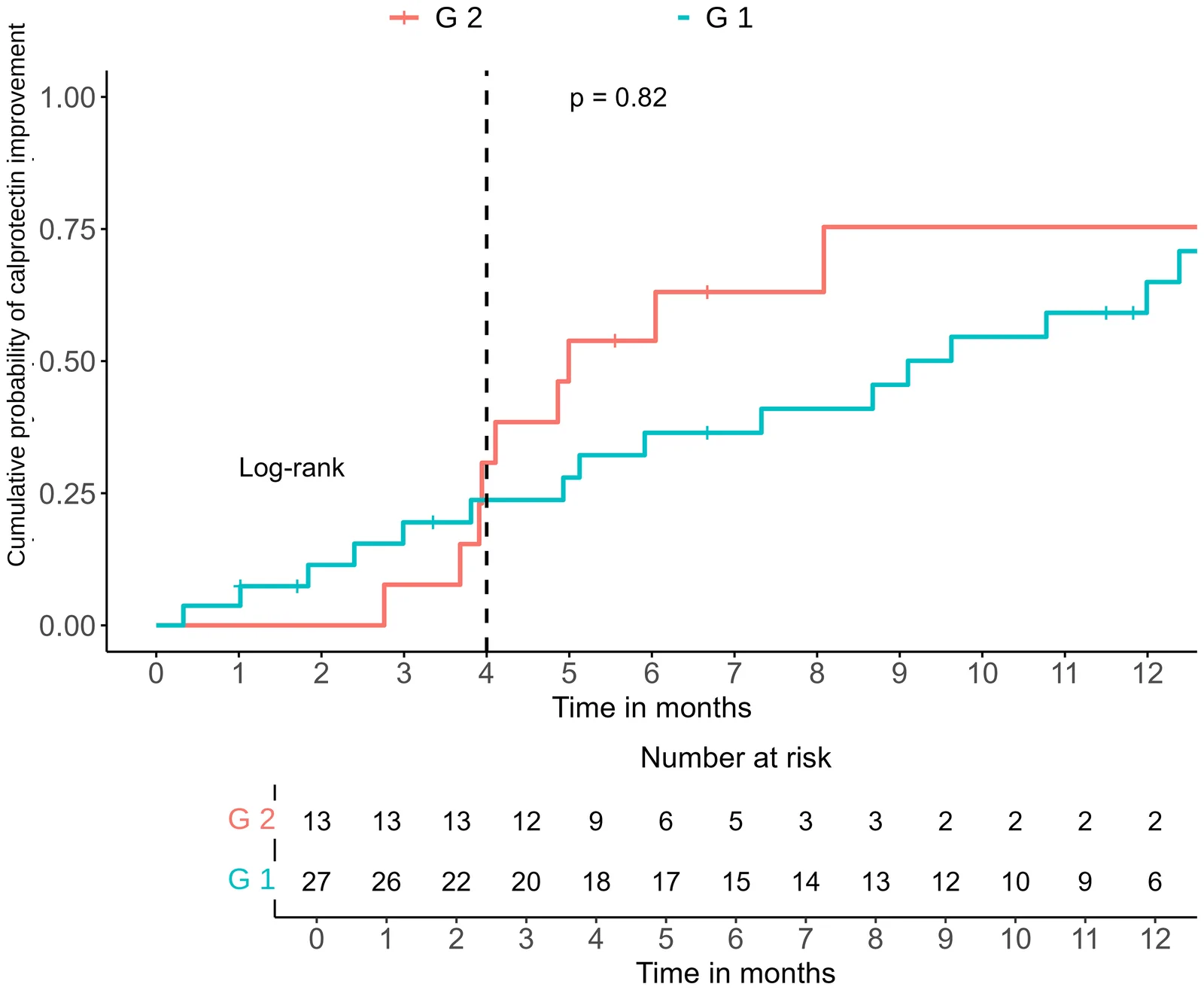
Cumulative incidence of calprotectin improvement according to CDED status. Kaplan–Meier curves show the cumulative incidence of calprotectin improvement during follow-up according to CDED status. Differences between groups were assessed using the log-rank test. No statistically significant differences were observed between groups (p=0.82). The lower panel shows the number of patients at risk at each time point. The vertical dashed line indicates the prespecified 4-month time point selected for cumulative incidence estimation.

Mucosal healing was achieved in 22/40 patients (55%): 15/27 (55.6%) in G1 and 7/13 (53.8%) in G2. Since mucosal healing was achieved at different time points during follow-up, cumulative incidence was assessed using Kaplan–Meier analysis. Kaplan–Meier analysis (**Figure 2**) showed no significant difference in the cumulative incidence of mucosal healing between G1 and G2 during follow-up (log-rank p = 0.54). At the prespecified 12-month time point, the cumulative incidence of mucosal healing was 18.2% (95% CI: 0.0–38.1%) in G2 and 16.0% (95% CI: 3.0–35.0%) in G1.

**Figure 2:**
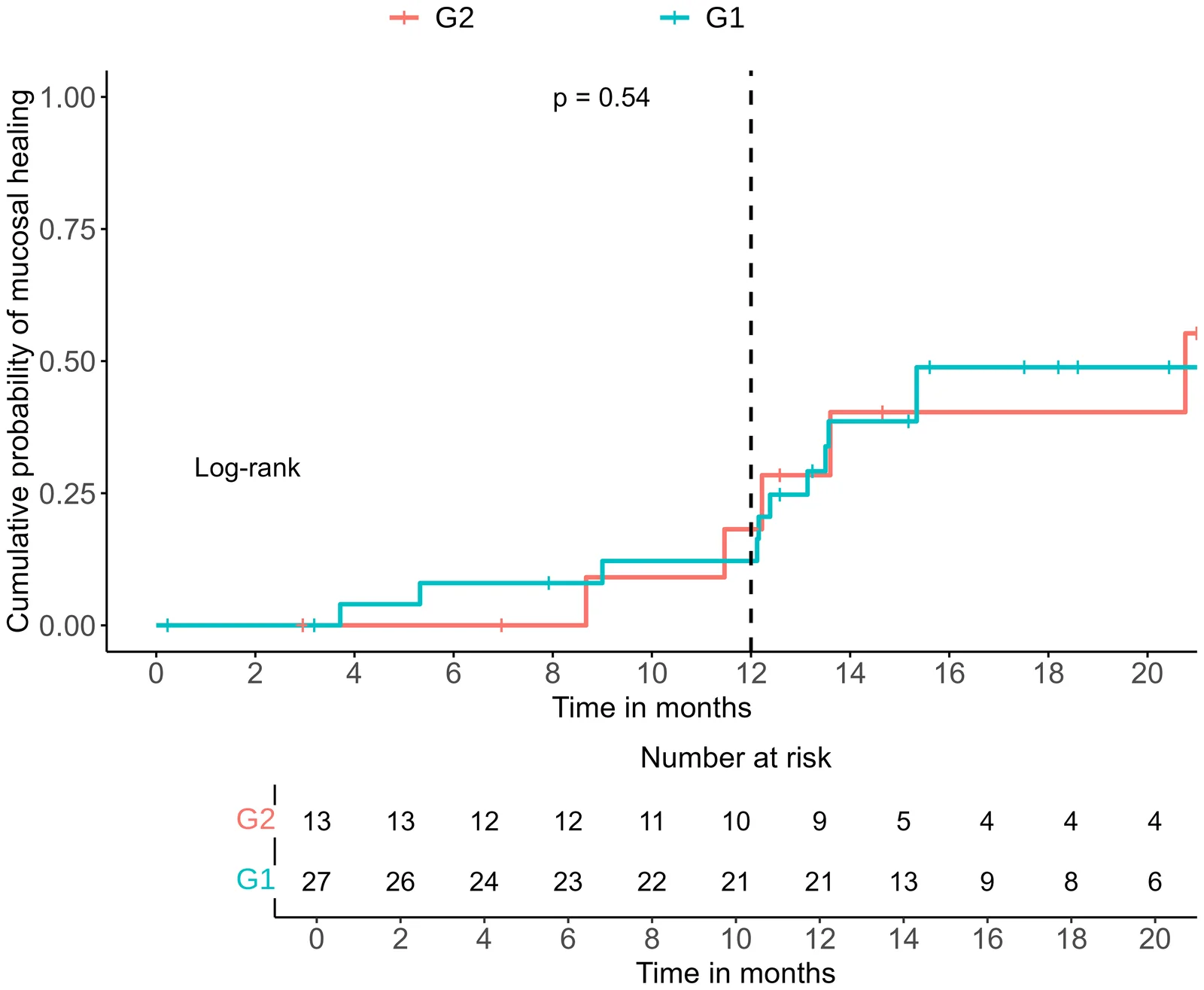
Cumulative incidence of Mucosal Healing according to CDED status. Kaplan–Meier curves show the cumulative incidence of mucosal healing during follow-up according to CDED status. Differences between groups were assessed using the log-rank test. No statistically significant differences were observed between groups (p=0.54). The lower panel shows the number of patients at risk at each time point. The vertical dashed line indicates the prespecified 12-month time point selected for cumulative incidence estimation.

To assess whether disease location modified the effect of CDED, patients were grouped into colonic-only disease (L2; n = 10) and ileal involvement (L1/L3/L4a/L4b; n = 30). Median regression models including a diet-by-location interaction term showed no statistically significant interaction for any of the evaluated outcomes: wPCDAI (p for interaction = 0.870), fecal calprotectin (p = 0.814), SES-CD (p = 0.390), ESR (p = 0.938), CRP (p = 0.105), albumin (p = 1.000), or BMI (p = 0.128). Thus, there was no evidence that disease location modified the effect of CDED on changes in clinical, biochemical, or endoscopic outcomes. Given the small number of patients with exclusively colonic disease (n = 10), these findings should be considered exploratory.

Mucosal response (≥50% reduction in SES-CD) was achieved in 13/13 (100%) patients in G2 and 25/27 (92.6%) in G1 (p = 0.99). Given the absence of difference and the potential ceiling effect, we evaluated absolute changes (delta) from baseline to last follow-up for wPCDAI, fecal calprotectin, SES-CD, ESR, CRP, albumin, and BMI. Most variables improved significantly within each group when comparing baseline and follow-up values (**Table 3**). When comparing the magnitude of change (Δ) between groups (**Figure 3**), the only statistically significant difference was observed for wPCDAI, with a greater reduction in G1 compared to G2 (p = 0.029).

**Table 3:** Baseline and follow-up clinical, biochemical, and endoscopic outcomes according to CDED.

Measurement, median (IQR)	Baseline			Follow-up			
	G1	G2	*p*	G1	*p* pre–post	G2	*p* pre–post
Fecal calprotectin median [IQR]	1000 (968.5–1022)	1000 (519–1000)	0.177	103 (62.5–275)	<0.001	130 (66–195)	<0.001
wPCDAI median [IQR]	45 (41–49)	42 (40–45)	0.083	5 (0–12)	<0.001	10 (5–15)	0.002
SES-CD median [IQR]	7 (6.5–15)	11 (8–11)	0.495	0 (0–3.5)	<0.001	3 (0–8)	0.034
ESR median [IQR]	36 (19–57)	18 (13–27)	0.051	13 (9–24)	<0.001	10 (9–11)	0.028
CRP median [IQR]	11.9 (5.5–33)	5 (3–12.4)	0.091	1 (0–3.5)	<0.001	1 (0–1)	0.007
Albumin median [IQR]	3.5 (3.1–3.8)	3.8 (3.7–4.0)	0.006	4.0 (3.8–4.2)	0.004	4.3 (4.0–4.4)	0.008
BMI median [IQR]	16.9 (14.85–18.15)	17.4 (15.2–22.0)	0.170	17.8 (16.7–20.2)	0.001	18.2 (15.7–23.2)	0.126

Baseline comparisons between the G1 and G2 groups were performed using the Wilcoxon rank-sum test. Baseline-to-follow-up comparisons within each group were performed using the paired Wilcoxon signed-rank test. A two-sided p value <0.05 was considered statistically significant.

wPCDAI, weighted Pediatric Crohn’s Disease Activity Index; SES-CD, Simple Endoscopic Score for Crohn’s Disease; ESR, erythrocyte sedimentation rate; CRP, C-reactive protein; BMI, body mass index; IQR, interquartile range.

**Figure 3:**
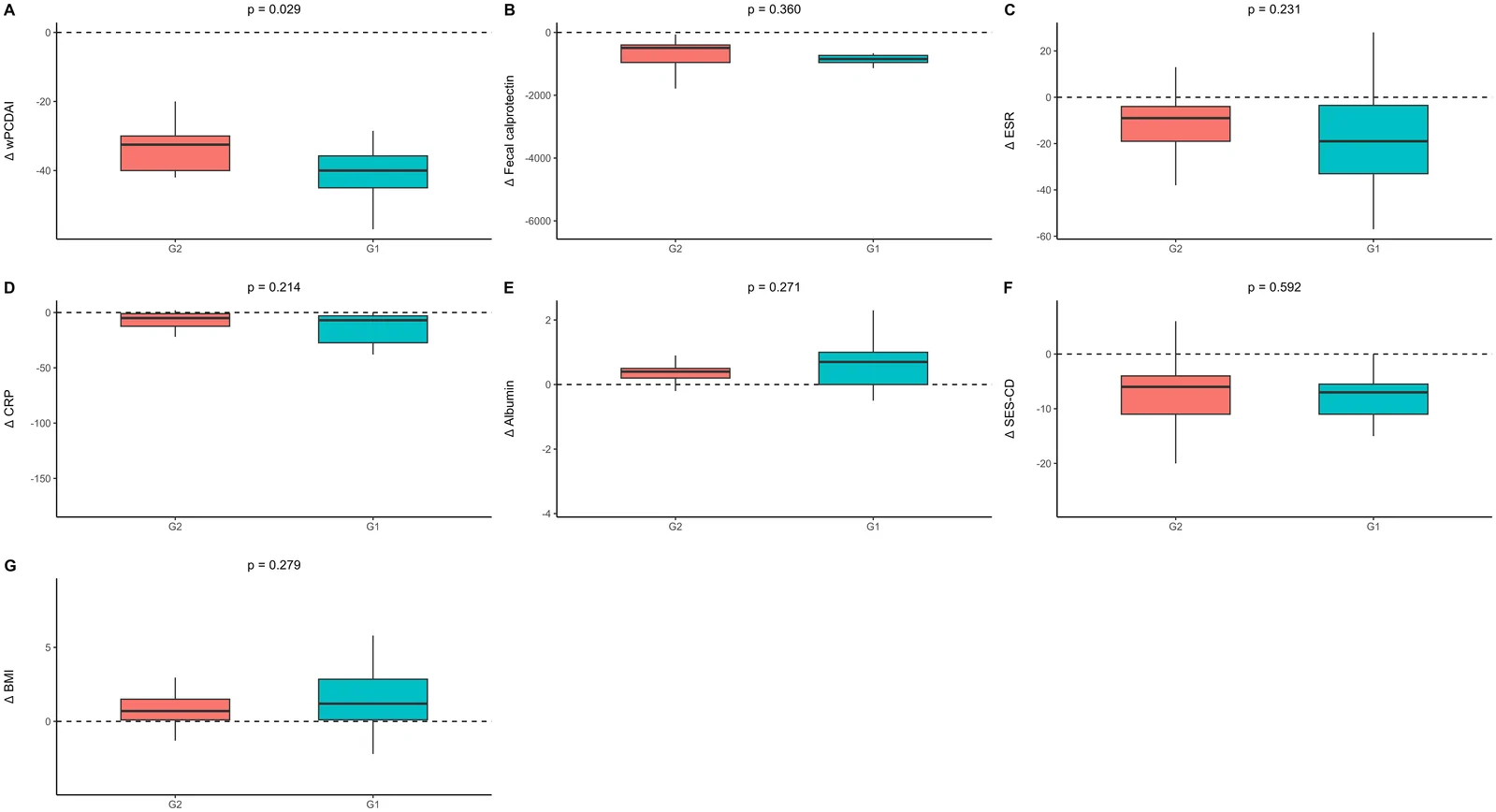
Changes in clinical, biochemical, and endoscopic outcomes according to CDED. **(A)** Weighted Pediatric Crohn’s Disease Activity Index (wPCDAI), **(B)** fecal calprotectin, **(C)** erythrocyte sedimentation rate (ESR), **(D)** C-reactive protein (CRP), **(E)** albumin, **(F)** Simple Endoscopic Score for Crohn’s Disease (SES-CD), and **(G)** body mass index (BMI). Box plots represent changes (Δ) from baseline to follow-up. The center line within each box represents the median, the lower and upper limits of the box represent the first and third quartiles (Q1 and Q3), respectively, and the whiskers extend to 1.5 times the interquartile range (IQR). The horizontal dashed line indicates no change (Δ = 0). Changes were calculated as follow-up minus baseline values. P values correspond to between-group comparisons of changes using the Wilcoxon rank-sum test.

## 4 Discussion

In this real-world pediatric cohort, adding CDED+PEN to first-line anti–TNF-α therapy was associated with a greater magnitude of clinical improvement, but not with faster or more frequent achievement of biochemical or endoscopic targets. The reduction in wPCDAI from baseline was significantly larger in G1 than in G2 (p = 0.029), and times to clinical response, clinical remission, and ESR/CRP normalization were consistently, though not significantly, shorter in G1. In contrast, the cumulative incidence of fecal calprotectin normalization was 23.7% in G1 versus 30.8% in G2 at 4 months (log-rank p = 0.82), and that of mucosal healing 16.0% versus 18.2% at 12 months (log-rank p = 0.54), with overlapping confidence intervals and no evidence of separation in either direction.

This dissociation between symptomatic and objective outcomes deserves emphasis rather than explanation away. Dietary therapy in CD has been most consistently linked to rapid symptom relief. In the randomized controlled trial by Levine et al., conducted in children with mild-to-moderate CD at centers in Israel and Canada and published in 2019, CDED+PEN achieved higher tolerability and sustained week-12 remission than EEN in 78 randomized patients (6). A comparable effect has been reported in adults in the open-label CDED-AD pilot trial by Yanai et al. (2022), in which 44 patients with mild-to-moderate disease were randomly assigned to CDED with or without PEN; in the intention-to-treat analysis, clinical remission at week 6 was achieved in 68% and 57% respectively (9). The consistency of this clinical effect across settings has been summarized in recent reviews (7, 8). Our data are compatible with that effect being preserved when the diet is added to a biologic; what they do not support is an incremental effect on the inflammatory burden already addressed by anti–TNF-α blockade. Two features of the cohort argue against dismissing the clinical signal as noise: patients in G2 had significantly higher baseline albumin, suggesting a less compromised phenotype that would be expected to bias comparisons against G1, and the wPCDAI difference persisted despite this. Conversely, the additional dietitian visits and inter-visit contact available only to G1 constitute a plausible non-dietary attention effect on a symptom-based index, which our design cannot exclude.

Our results extend, rather than replicate, the available literature on CDED as an adjunct to biologic therapy. Previous real-world experience has consistently reported feasibility and clinical benefit in this setting, both at disease onset and in patients refractory to or losing response to biologics, with sustained remission rates of 60–87% and concomitant improvement in fecal calprotectin and albumin (14, 15). In the largest pediatric real-life series to date, an early clinical response by week 4 independently predicted both clinical remission and fecal calprotectin normalization, whereas a higher inflammatory burden and extraintestinal manifestations were associated with lower response rates (15). Where CDED+PEN has been compared directly against EEN alongside infliximab, clinical and endoscopic remission rates were equivalent, with an advantage for CDED in BMI Z-score and week-12 fecal calprotectin (13). Our cohort differs in that the comparator was biologic therapy without structured dietary intervention, which isolates the incremental contribution of the diet rather than comparing two nutritional strategies against each other. Under that design, the incremental contribution appears to be clinical rather than biochemical. This does not contradict the earlier reports, most of which lacked a diet-free biologic comparator, but it raises the possibility that part of the biochemical benefit attributed to CDED in single-arm cohorts reflects the concurrent biologic.

A biological rationale for a genuine adjunctive effect remains plausible and should not be discarded on the basis of a cohort of this size. CDED removes dietary components that promote dysbiosis and barrier impairment while favoring a microbiome shift—characteristically a reduction in Proteobacteria and an expansion of Firmicutes—together with changes in tryptophan and related metabolite pathways associated with diet-induced remission (5). In adults with active CD, EEN combined with biologics has been reported to yield higher endoscopic response and remission rates than biologics alone, alongside improved body composition, optimized biologic pharmacokinetics, and reduced inflammatory cytokine levels (16). Our findings do not confirm that this synergy extends to endoscopic outcomes in children receiving CDED+PEN, but a cohort of 40 patients with 13 controls is underpowered to exclude a moderate effect, and the wide confidence intervals around our Kaplan–Meier estimates reflect this directly.

The absence of a difference in endoscopic outcomes is best interpreted in light of the timeframe rather than as evidence of no effect. Under STRIDE-II (10), normalization of fecal biomarkers is a short-term therapeutic target whereas endoscopic healing is a long-term one; our rates and timing are broadly consistent with pediatric anti-TNF-α cohorts, in which mucosal and histologic healing accrue gradually and in a substantial but incomplete proportion of patients (17, 18). A recent systematic review of nutritional interventions during the maintenance phase of pediatric CD likewise found the evidence linking dietary therapy to mucosal-barrier and endoscopic outcomes to be limited and heterogeneous, with complete healing frequently unachieved within standard induction windows (19). Detecting an incremental endoscopic effect of diet added to a biologic would require both longer follow-up and protocolized endoscopic timing, neither of which a real-world retrospective design provides. The heterogeneous monitoring intervals of routine care, together with the known intra-individual variability of fecal calprotectin (20, 21), further limit the precision of our time-to-event estimates.

Clinically, these findings support a narrower indication than is sometimes assumed. Adjunctive CDED+PEN appears reasonable when the goal is faster symptomatic improvement during biologic induction—a period in which children remain symptomatic while awaiting pharmacological response—but our data do not support the expectation that it will accelerate biomarker normalization or endoscopic healing. Given the adherence burden and the multidisciplinary resources the diet requires (5, 18), that distinction matters when counseling families, and the diet should not be positioned as a substitute for adequate optimization of biologic therapy. The decision to add CDED+PEN is therefore best individualized.

### 4.1 Limitations

This study has several limitations, including its retrospective, single-center design, modest sample size (particularly in the monotherapy group, n=13), and the decision to add dietary therapy was not randomized, which may have introduced selection bias. The real-world setting entailed heterogeneous monitoring intervals and variable timing of fecal calprotectin measurements, and its known intra-individual variability (20, 21) may have influenced time-to-event estimates. Additionally, systematic data on anti-TNF dose optimization and therapeutic drug levels were not available for all patients, which may have influenced outcomes and limits the granularity of our analysis. The CDED requires intensive multidisciplinary support and may not be generalizable to populations with different dietary habits or healthcare infrastructures (5, 18). Although both groups received structured in-person follow-up with the pediatric gastroenterologist according to institutional protocols, patients in G1 had additional in-person visits with an IBD-specialized dietitian and access to telephone or email support between visits. This additional attention and multidisciplinary support may have introduced a non-dietary attention bias, potentially contributing to the observed trends toward earlier improvement in G1. Residual confounding by unmeasured variables cannot be excluded. Despite these limitations, our findings provide real-world evidence supporting the potential benefit of adjunctive CDED+PEN, warranting confirmation in prospective, adequately powered studies.

## 5 Conclusion

In this real-world pediatric cohort, adding CDED+PEN to first-line anti–TNF-α therapy was associated with a significantly greater reduction in wPCDAI, but not with improved cumulative incidence of fecal calprotectin normalization or mucosal healing. The incremental value of adjunctive dietary therapy in children already receiving a biologic may therefore lie in earlier symptomatic control rather than in accelerated resolution of objective inflammation, although the sample size does not exclude a moderate effect on the latter. These hypothesis-generating findings warrant confirmation in larger prospective studies aligned with STRIDE-II targets.

## Data Availability

The raw data supporting the conclusions of this article will be made available by the authors, without undue reservation.
